# Negative vaccine sentiments on South African social media platforms before the COVID-19 pandemic: a mixed methods study

**DOI:** 10.3389/frhs.2025.1578992

**Published:** 2025-05-30

**Authors:** Michelle M. Matsangaise, Rosemary J. Burnett, Zeenat Ismail, Johanna C. Meyer

**Affiliations:** ^1^ Department of Public Health Pharmacy and Management, School of Pharmacy, Sefako Makgatho Health Sciences University, Pretoria, South Africa; ^2^ South African Vaccination and Immunisation Centre, Sefako Makgatho Health Sciences University, Pretoria, South Africa; ^3^ Department of Virology, School of Medicine, Sefako Makgatho Health Sciences University, Pretoria, South Africa,

**Keywords:** South Africa, vaccine hesitancy, vaccine sentiments, social media, pre-pandemic, human papillomavirus

## Abstract

**Introduction:**

During the pre-pandemic era, negative vaccine sentiments did not feature in South African publications reporting on infant vaccination uptake. In contrast, vaccine hesitancy is an established driver of suboptimal COVID-19 vaccine uptake in South Africa, suggesting that the COVID-19 pandemic increased vaccine hesitancy in South Africa. This study used data from a social media tracking project to investigate vaccine sentiment expressed on South African social media platforms in the pre-pandemic era.

**Methods:**

This mixed-methods study analysed South African social media [Twitter (now X); online news forums; microblogs] posts mentioning vaccine-related words from 1 December 2016–31 May 2017. Content analysis was used to assign vaccine sentiment, and thereafter a step-wise thematic content analysis of negative sentiment posts was conducted using NVivo12®.

**Results:**

Of 10,997 posts about human vaccines, 16.2% expressed negative vaccine sentiments. Specific vaccines were discussed in 35.9% of posts, with human papillomavirus (HPV) vaccines attracting the most negative sentiments (31.9% of all negative posts). The majority of negative posts included links to articles emanating from other countries, predominantly the USA. Five themes were identified: Vaccine safety; autism; vaccine effectiveness; conspiracy theories; and philosophical/religious objections.

**Discussion:**

Relatively high levels of pre-existing negative sentiments toward vaccines were expressed in the pre-pandemic era, with HPV vaccines attracting the most negative comments. These results provide a baseline for comparison to post-pandemic social media studies and may prove useful for measuring the impact in South Africa of global policies introduced to limit the spread of vaccine mis- and disinformation.

## 1 Introduction

In 2018, the rapid dissemination of vaccine mis- and disinformation, spread mainly through social media, was identified through research conducted by the Vaccine Confidence Project™ (https://www.vaccineconfidence.org/) as a major threat to global public health (1). Then in 2019, based on the alarming increase in outbreaks of vaccine-preventable diseases (VPDs) that had previously been eliminated in mainly high-income countries, the World Health Organization (WHO) declared vaccine hesitancy, defined as “the reluctance or refusal to vaccinate despite the availability of vaccines” as one of the top 10 threats to global public health (2). However, it was in 2020/2021 that vaccine hesitancy captured the imagination of the general public globally, as a result of the social media-driven “infodemic” that undermined efforts to control the coronavirus disease 2019 (COVID-19) pandemic (3–5).

In South Africa, infant vaccination services are provided free of charge in the public sector (6). However, 16% of the South African population access healthcare, including vaccination services, at high cost through the private sector (7). Negative sentiments toward vaccines did not often feature prominently in published South African surveys and qualitative research conducted on vaccination uptake in the pre-pandemic era. When it did feature prominently as a driver of low vaccination coverage, it was largely confined to research on the human papillomavirus (HPV) vaccine (8–12), most of which was conducted online (9, 11, 12). While one study reported that 6.7% of vaccination programme managers perceived vaccine hesitancy (phrased as “resistance from parents and anti-immunisation rumours”) as a key challenge preventing the achievement of high routine infant vaccination coverage (13), there was little or no evidence of negative sentiments toward vaccines from face-to-face household vaccination coverage surveys (6, 14–18). This evidence ranged from 0.4% (1/276) of participants who “have no faith in vaccines” (1.5% of the under-vaccinated) (14) to 3.1% (116/3,705) of participants who “lack motivation” to vaccinate their children (13.5% of the under-vaccinated) (17). However, none of these face-to-face household vaccine coverage surveys included caregivers living in exclusive communities (i.e., high property-value walled or gated communities and security complexes (19), as in-person access to these communities has proven to be impossible (6, 14). It is highly likely that these exclusive communities have higher rates of internet access than low-income communities (14). While data on the extent of South Africans living in gated communities are lacking, it is also highly likely that the 16% of South Africans who can afford private healthcare (7), can afford internet access. Taken together, this may explain discrepancies in reports of negative vaccine sentiment between online surveys and face-to-face surveys conducted in South Africa during the pre-pandemic era. In addition, while all surveys inherently suffer from bias, surveys using face-to-face data collection have been shown to suffer more from social desirability bias than online surveys (20), which may result in under-reporting of vaccine hesitancy in household surveys.

In contrast to the pre-pandemic era, negative sentiments toward COVID-19 vaccines featured in all South African COVID-19 vaccine acceptance and uptake surveys (21–26), and in multinational vaccine confidence surveys that include South Africa (27–30). There is also global evidence including from South Africa, suggesting that negative COVID-19 vaccine sentiments have undermined confidence in vaccines routinely received during childhood (27, 28, 31). For example, the United Nations Children's Fund estimates that the COVID-19 pandemic resulted in a 30% decline in confidence in childhood vaccines in South Africa, with only 62% of the population being confident that vaccines are important for children (31), suggesting that the COVID-19 pandemic dramatically increased vaccine hesitancy in South Africa. In the absence of published evidence of vaccine sentiment expressed on South African social media platforms before the pandemic, we used data from a social media tracking project conducted to investigate vaccine sentiment expressed on South African social media platforms in the pre-pandemic era. This baseline is essential for measuring the impact in South Africa of global policies introduced by social media platforms during the pandemic, to limit the spread of vaccine mis- and disinformation (4). It can also be used to assess the impact of post-pandemic enhanced vaccine communication strategies aimed at increasing public confidence in, and uptake of, routine childhood vaccines.

## 2 Materials and methods

### 2.1 Study design and study population

This mixed-methods study was based on social media posts emanating from South African IP addresses. Data were collected from 1 December 2016 to 31 May 2017, using Pulsar® software, a commercially available online “social listening” tool (https://www.pulsarplatform.com/). Facebook was excluded from the project because of its privacy rules; thus, the project was confined to Twitter (now X), online news forums and other microblogs. No sampling was conducted; all posts with South African IP addresses in the public domain were included. South Africa has 11 official written languages (sign language is the 12th official language); however, the search terms were confined to keywords in English and Afrikaans, based on previous findings of no hits when searching for Internet content using the terms for vaccination in the other official languages (32). This search strategy is supported by previous research on South Africans whose home languages were isiZulu, siSwati, Sepedi, Tshivenda, and Xitsonga, which reported that “most of the respondents prefer using the English language on social media” (33). Because South African social media users tend to use a mixture of languages (33), language was categorised according to the predominant language used in each post. The following Boolean search string was used: *Vaccination OR vaccinate OR vaccine OR vaccinations OR vaccinates OR vaccines OR vaccinated OR #vaccination OR #vaccinate OR #vaccine OR #vaccinations OR #vaccinates OR #vaccines OR #vaccinated OR inenting OR entstof OR entstowwe OR inentings OR ingeënt OR #ingeënt OR #inenting OR #entstof OR #entstowwe OR #inentings*.

### 2.2 Data collection and sentiment coding

While Pulsar® software is designed to code sentiment as positive, neutral or negative, during piloting it was decided that given the complexity of vaccine hesitancy that resulted in frequent misclassification, vaccine sentiment assignments should be manually recoded to ensure validity. Thus, for this study, vaccine sentiment was checked and recoded in two phases, using a content analysis approach associated with qualitative research to generate quantifiable data. In the first phase, MMM and a research assistant reviewed each post, and manually changed the software-assigned sentiment if they both agreed on the change. Where there was disagreement between the two, JCM made the decision on sentiment allocation. In the second phase, RJB reviewed all posts, and where there was disagreement with the sentiments allocated in the first phase, ZI made the final decision. In total, 85.4% (9,386/10,997) of the sentiments were manually reclassified. Of all posts classified as positive, negative and neutral after validation, respectively 88.5% (7,400/8,366), 70.1% (1,252/1,785) and 86.8% (734/846) had been manually reclassified. “Negative” was assigned to any post suggesting that vaccination may or should be delayed or refused (i.e., any concerns about vaccination, such as concerns about or questioning of vaccine safety, vaccine ingredients, vaccine effectiveness, or any other concern that suggested that vaccination may or should be delayed or refused). “Positive” was assigned to any post that promoted vaccination in any way including optimism about new vaccines under development. “Neutral” was assigned to any post where no clear tone or position could be discerned (i.e., posts that neither promoted nor cast doubt on the value of vaccination). In addition, data were collected on the source of the posts, and the contents of the posts.

### 2.3 Quantitative data analysis

Data were downloaded from Pulsar® in Microsoft Excel® (Microsoft Office, USA) format, and imported to Epi Info™ 7.2.5.0 (Centers for Disease Control and Prevention, USA) for descriptive statistical analysis. Furthermore, the Microsoft Excel data were also imported into Microsoft Access® (Microsoft Office, USA) for analysis of the posts to identify any vaccines that were mentioned. For each vaccine, an Access query was run using the words listed in Table 1 as search criteria with an asterisk on either side (i.e., the Access query format used to identify a word anywhere within the text).

**Table 1:** Frequency distribution of vaccines mentioned, stratified by sentiment (*n* = 3,950).^a^

Vaccine mentioned	Sentiments							
	Positive		Neutral		Negative		Total	
	*n*	%^b^	*n*	%^b^	*n*	%^b^	*n*	%
Measles	726	94.5	12	1.6	30	3.9	768	19.4
HIV	711	94.2	26	3.4	18	2.4	755	19.1
Flu/influenza	421	76.5	26	4.7	103	18.7	550	13.9
HPV/Gardasil/Cervarix	324	66.1	43	8.8	123	25.1	490	12.4
Ebola	339	97.4	5	1.4	4	1.1	348	8.8
Meningo	315	96.9	3	0.9	7	2.2	325	8.2
Polio	230	74.0	27	8.7	54	17.4	311	7.9
Malaria	77	95.1	2	2.5	2	2.5	81	2.1
Rotavirus	66	97.1	1	1.5	1	1.5	68	1.7
Cholera	56	100.0	0	0.0	0	0.0	56	1.4
Mumps	45	81.8	3	5.5	7	12.7	55	1.4
Pertussis/whooping cough	45	83.3	3	5.6	6	11.1	54	1.4
MMR	26	54.2	3	6.3	19	39.6	48	1.2
BCG/TB/tuberculosis	32	72.7	7	15.9	5	11.4	44	1.1
Varicella/chickenpox	19	52.8	3	8.3	14	38.9	36	0.9
Hepatitis	22	62.9	9	25.7	4	11.4	35	0.9
Pneumococcal	22	81.5	5	18.5	0	0.0	27	0.7
Shingles/herpes zoster	14	51.9	1	3.7	12	44.4	27	0.7
Zika	24	88.9	1	3.7	2	7.4	27	0.7
Tetanus	18	69.2	4	15.4	4	15.4	26	0.7
Diphtheria	24	92.3	1	3.8	1	3.8	26	0.7
Rubella/German measles	19	82.6	3	13.0	1	4.3	23	0.6
*Haemophilus influenzae* type B	5	83.3	1	16.7	0	0.0	6	0.2

^a^ Some posts mention more than 1 vaccine, thus the total of all mentions >3,950.

^b^ Denominators based on the total number of posts mentioning each specific vaccine.

### 2.4 Qualitative data analysis of negative sentiment

The contents of all posts with negative sentiment codes were imported into NVivo12®, a qualitative data analysis computer software package. A step-wise thematic content analysis of the data was conducted by MMM and JCM. Coding of the data commenced, following detailed reading of the posts in NVivo. Data were coded into nodes by MMM and re-coded by JCM to ensure the trustworthiness of the coding process. Continuous discussions between the two authors followed until consensus was reached about the coding, which further ensured the dependability of the data. Nodes were grouped into categories and sub-categories. Categories were then linked to one another forming overarching themes with sub-themes that depicted shared anti-vaccination sentiments.

### 2.5 Ethical considerations

This study was approved by the Sefako Makgatho University Research Ethics Committee (SMUREC/P/60/2018:PG) prior to data collection. All data were available in the public domain, and where personal identifiers of the social media account holders were evident, these data were neither collected nor reported in the results.

## 3 Results

### 3.1 Overall description of vaccine-related posts

A total of 11,111 posts mentioning a keyword were collected during the study period. After removing posts referring to animal vaccinations and the rock band “The Vaccines”, 10,997 records remained. Of these, 97.9% (10,766/10,997) were written mainly in English. The vast majority of the posts were on Twitter (now X), and expressed positive sentiments toward vaccines. See Table 2 for further details.

**Table 2:** Frequency of sources of vaccine-related posts, stratified by sentiment (*n* = 10,997).

Source	Sentiments							
	Positive		Neutral		Negative		Total	
	*n*	%	*n*	%	*n*	%	*N*	%
Twitter	6,994	75.4	605	6.5	1,672	18.0	9,271	84.3
News	1,100	86.5	155	12.2	16	1.3	1,271	11.6
Forums	250	59.0	81	19.1	93	21.9	424	3.9
Blogs	22	71.0	5	16.1	4	12.9	31	0.3
Totals	8,366	76.1	846	7.7	1,785	16.2	10,997	

Of all posts, 35.9% (3,950/10,997) mentioned one or more vaccine/s listed in Table 1. Of the posts mentioning specific vaccines, 85.7% (3,387/3,950) were positive, 4.5% (178/3,950) were neutral, and 9.7% (385/3,950) were negative. Vaccines which attracted the most positive sentiments were those against measles and human immunodeficiency virus (HIV) infection, with respectively 21.4% (726/3,387) and 21.0% (711/3,387) of vaccine-specific positive posts. Vaccines which attracted the most negative sentiments were those against HPV infection and influenza, with respectively 31.9% (123/385) and 26.8% (103/385) of vaccine-specific negative posts. See Table 3 for further details.

**Table 3:** Frequency distribution of sentiments stratified by vaccines mentioned (*n* = 3,950).^a^

Vaccine mentioned	Sentiments							
	Positive (*n* = 3,387)		Neutral (*n* = 178)		Negative (*n* = 385)		Total	
	*n*	%^b^	*n*	%^b^	*n*	%^b^	*n*	%
Measles	726	21.4	12	6.7	30	7.8	768	19.4
HIV	711	21.0	26	14.6	18	4.7	755	19.1
Flu/influenza	421	12.4	26	14.6	103	26.8	550	13.9
HPV/Gardasil/Cervarix	324	9.6	43	24.2	123	31.9	490	12.4
Ebola	339	10.0	5	2.8	4	1.0	348	8.8
Meningo	315	9.3	3	1.7	7	1.8	325	8.2
Polio	230	6.8	27	15.2	54	14.0	311	7.9
Malaria	77	2.3	2	1.1	2	0.5	81	2.1
Rotavirus	66	1.9	1	0.6	1	0.3	68	1.7
Cholera	56	1.7	0	0.0	0	0.0	56	1.4
Mumps	45	1.3	3	1.7	7	1.8	55	1.4
Pertussis/whooping cough	45	1.3	3	1.7	6	1.6	54	1.4
MMR	26	0.8	3	1.7	19	4.9	48	1.2
BCG/TB/tuberculosis	32	0.9	7	3.9	5	1.3	44	1.1
Varicella/chickenpox	19	0.6	3	1.7	14	3.6	36	0.9
Hepatitis	22	0.6	9	5.1	4	1.0	35	0.9
Pneumococcal	22	0.6	5	2.8	0	0.0	27	0.7
Shingles/herpes zoster	14	0.4	1	0.6	12	3.1	27	0.7
Zika	24	0.7	1	0.6	2	0.5	27	0.7
Tetanus	18	0.5	4	2.2	4	1.0	26	0.7
Diphtheria	24	0.7	1	0.6	1	0.3	26	0.7
Rubella/German measles	19	0.6	3	1.7	1	0.3	23	0.6
Haemophilus type B	5	0.1	1	0.6	0	0.0	6	0.2

^a^ Some posts mention more than 1 vaccine, thus the total of all mentions >3,950.

^b^ Denominators based on the total number of each sentiment type.

### 3.2 Results of thematic analysis of negative sentiments

The majority of posts expressing negative sentiments included links to articles emanating from other countries, predominantly the USA. However, data analysis was not conducted on these articles, thus the results reported here are confined to posts emanating from South Africa. Thematic analysis generated five overarching themes which are listed in Table 4 together with their defining properties. Where sub-themes emerged within some of the themes, these sometimes overlapped with other sub-themes within the same theme or other themes.

**Table 4:** Summary of main themes and their defining properties.

Main theme	Defining properties of posts
Vaccine safety concerns	Infer that vaccines *per se* cause serious adverse outcomes/diseases/death
	Infer that vaccines contain toxic/harmful cancer-causing additives
	Infer that receiving multiple vaccines simultaneously causes serious adverse outcomes/diseases/death
Autism concerns	Blame toxic/harmful additives in vaccines for causing autism
	Blame receiving multiple vaccines simultaneously as a cause of autism
	Accuse a named/unnamed powerful person/organisation of targeting a specified population with vaccines that cause autism
Concerns about vaccine effectiveness	Infer that vaccines are ineffective because vaccine preventable diseases occur in vaccinated people
	Infer that vaccines are deliberately designed to be ineffective
Conspiracy theories	Accuse a named/unnamed powerful person/organisation for developing/using vaccines to harm a specified population
	Accuse a named/unnamed pharmaceutical company of manipulating data to make vaccines seem effective
	Accuse a named/unnamed powerful person/organisation for manufacturing/promoting vaccines solely for profit
Philosophical/religious objections	Infer that vaccines contain substances that conflict with personal or religious beliefs
	Infer that vaccination *per se* is a violation of human rights

#### 3.2.1 Vaccine safety concerns

The overarching theme on concerns about vaccine safety included various sub-themes. One sub-theme centred around the perception that vaccines cause diseases, including autoimmune disorders and the diseases against which they are supposed to protect. In particular, it was believed that many children have died from HPV vaccination, and that the HPV vaccine causes autoimmune diseases, ovarian failure, myocarditis, clotting and severe pain. It was also believed that the HPV vaccine causes cervical cancer, and the influenza vaccine causes influenza. Within this sub-theme was the common belief that vaccines enhance the spread of infectious diseases. Another sub-theme was that vaccination results in the development of adverse effects affecting the brain. In addition to autism, these included obsessive compulsive disorder, anorexia nervosa, a host of other brain and neurological disorders and death, with the HPV vaccine being commonly blamed for convulsions, neuroinflammation, encephalopathy, brain damage and paralysis. A third sub-theme centred on the belief that vaccines contain additives which predispose recipients to developing cancer. This sub-theme was linked to a fourth sub-theme centred on vaccine additives which were often labelled as “harmful” and “toxic”, particularly regarding HPV vaccines. Mentions of ingredients such as the mercury-based preservative thimerosal (also referred to as mercury in many posts) were common, with many believing that mercury in vaccines is responsible for causing neurological disorders. Another additive, aluminium, was implicated in causing genetic changes that are passed on from one generation to the next. See Table 5 for examples of these posts.

**Table 5:** Examples of quotes expressing *vaccine safety concerns.*

Source	Verbatim quotes
Twitter^a^	By 2013 Gardasil use has resulted in over 30,000 serious injuries and over 150 death (VAERS)
Twitter	…death was believed to have been caused by eight simultaneous vaccinations
Twitter	Did you know that the Gardasil Vaccine is linked to Neuroinflammation and Autoimmune Reactions?
Twitter	People vaccinated against #Flu 3 years in a row are @ higher risk of catching the flu
Twitter	Countless teenage girls suffer paralysis, blood clots, brain damage and chronic pain from force-vaccination of Gardasil's HPV.
Twitter	…just seems so unnatural to inject her with chemicals. &; also vaccination injury/getting sick from the vaccine itself
Twitter	DO NOT VACCINATE!!! The sh** is laced with cancer inducing additives
Twitter	13-Year-Old Boy Permanently Disabled From Chicken Pox Vaccine Wins His Case In Vaccine Court
Twitter	CDC's new vaccine schedule adds toxic, ovary destroying HPV #vaccines to recommendations
Twitter^b^	The Amish, who don’t get vaccinated, rarely get autism, cancer, or heart disease—coincidence?

^a^ Twitter is now known as X.

^b^ Overlaps with autism theme.

#### 3.2.2 Autism concerns

Linked to and overlapping with the vaccine safety and conspiracy theory themes, but emerging as a theme on its own, was the belief that vaccines cause autism. Several posts overlapped with the vaccine safety sub-theme of brain disorders, since they conveyed the belief that vaccine additives such as mercury resulted in the development of autism and autism spectrum disorders, while others conveyed the belief that the “excessive” quantity of vaccines are responsible. A few posts were linked to the conspiracy theory theme, suggesting that vaccines were a government plot to give Black children autism. Some posts were about the belief that a loved one developed autism after vaccination, while many made reference to claims by former USA President Donald Trump, where he had expressed his belief in a possible link between what he termed “monstrous combined vaccines” and autism. See Table 6 for examples of these posts.

**Table 6:** Examples of quotes expressing concerns about autism.

Source	Verbatim quotes
Twitter^a^	Egyptian study confirms autism is caused by mercury in vaccines
Twitter	Vaccination show clear signs of causing Autism. As a relative of a child who has this; it began after vaccinations. Concerns me
Twitter	A study says @Autism is out of control-a 78% increase in 10 years. Stop giving monstrous combined vaccinations
Twitter	What Did You Expect From The Vaccines? That's right-what did you expect?! No, thanks-I don't want no goddamn autism!
Twitter	Autism epidemic is real, and excessive vaccinations are the cause
Twitter	128 Research Papers Supporting the Vaccine/Autism Link
Twitter^b^	The Government is Using Vaccines to Give Black Kids Autism…can someone please share a light on this.its disturbing
Twitter	Italian Court Rules #Vaccines Really Do Cause #Autism
Twitter	RFKennedy is not anti-vaccine. 10+ scientific studies linking vaccines&autism. Did U do ANY *research* before writing your story?
Twitter	Trump's pick for Secretary of #Health and Human Services backs the #vaccine-autism connection

^a^ Twitter is now known as X.

^b^ Overlaps with conspiracy theory theme.

#### 3.2.3 Concerns about vaccine effectiveness

The anti-vaccination conversational landscape on the social media platforms in this study showed a general lack of confidence in the effectiveness of vaccines. Several posts conveyed doubts about vaccine effectiveness, with the HPV vaccine being called “dangerously ineffective”, and questioned why cases of vaccine-preventable diseases still occurred in people who had been vaccinated. Examples include contracting meningitis despite receiving the meningitis vaccine, and measles and mumps outbreaks where some cases had been vaccinated. Some posts expressed views that were linked to conspiracy theories. For example, one post hypothesised that the HIV vaccine was purposefully designed to be ineffective and was meant to encourage risky sexual behaviour. See Table 7 for examples of these posts.

**Table 7:** Examples of quotes expressing concerns about vaccine effectiveness.

Source	Verbatim quotes
Blog	Those who were vaccinated for the flu had 5.5 times more respiratory illness than those who were not vaccinated. Other studies show that repeated annual flu vaccines decrease their effectiveness
Twitter^a^	Shocking vaccine study finds that teens are being wildly overdosed with multiple HPV injections that do NOTHING to prevent
Twitter	My question is, how can unvaccinated kids be a problem to vaccinated kids? If vaccinations work
Twitter	If the vaccine in question is so weak that a small fringe group of people not vaccinated could pose any real threat to those vaccinated, then the issue is with the vaccine's inefficiency not the anti-vaxxers
Twitter	What if they see that AIDS is not infecting enough people then introduce an ineffective vaccine; to encourage risky sexual behaviour?
Twitter	Are VACCINES Really Effective? Vaccine Failures Keep Mounting
Twitter	Cervical cancer is killing African American women at twice the previously reported rate in spite of the HPV vaccine …
Twitter^b^	Vaccine fraud exposed: Measles and mumps making a huge comeback because vaccines are designed to fail, say Merck
Twitter^c^	Everyday is a battle—Teenager's Life Is Forever Ruined by the Dangerously Ineffective HPV Vaccine
Twitter	vaccines dont work—we are not in favor of vaccinating human beings or animals

^a^ Twitter is now known as X.

^b^ Overlaps with autism theme.

^c^ Overlaps with vaccine safety theme.

#### 3.2.4 Conspiracy theories

Conspiracy theories around government organisations, health boards and the pharmaceutical industry emerged as a recurring theme. Health regulatory boards such as the CDC and the USA Food and Drug Administration were often accused of withholding information on vaccine adverse effects and instead overinflating positive vaccine safety information. Many posts centred on racial motives, with phrases being used such as “germ warfare against black kids,” “depopulate the world,” “biological warfare” and “medical genocide” targeted at Africans. Some posts centred on Bill Gates, suggesting that he is planning on introducing “a new deadly vaccine” to South Africa, and that he is funding vaccination programmes “which would lead to pandemics”. Conspiracy theories around pharmaceutical companies centred on the common belief that vaccine manufacturing companies do so primarily for financial gain. These posts included calling vaccine programmes a “toxic scam so Big Pharma can make money,” and the opinion that pharmaceutical companies manipulate scientific data to make vaccines seem effective in order to make profit. See Table 8 for examples of these posts.

**Table 8:** Examples of quotes expressing conspiracy theories.

Source	Verbatim quotes
Blog	The fact is that vaccines are a $30 billion dollar a year industry, and those who benefit from it are going to do whatever they can to protect their own interests
Twitter^a^^,^^b^	Been telling y'all about the UN- they also infected people with the Ebola virus via a “vaccine” for free food#WokeBaby
Twitter^b^	Vaccines are full of toxins and carcinogens, including fetal tissue. The Elite have admitted that vaccines are being used for depopulation
Twitter	In America they infected African Americans with syphilis under the pretence it was a vaccination so imagine what they have done in Africa?
Twitter	American College of Pediatricians warns about toxic effects of Gardasil vaccine; sounds alarm over massive scientific fraud that concealed toxic effects
Twitter	So the US government supported the Apartheid regime & funded Basson's “scientific” projects & it's running a vaccine “trial” on Blacks
Twitter	Testing HIV/AIDS Vaccine over 4yrs as if we Don't know that the Cure is Available. Just Generating Large Profits for Pharmaceuticals.
Twitter^b^	Doctors murdered after discovering cancer enzymes in vaccines
Twitter^b^	Please do some deep Research on EUGENICS my Sister. Viruses, Sterilisation, Abortion, Vaccines, etc. Population Control
Twitter^b^	Same Bill Gates that pushes mosquito vaccines and depopulation agendas now celebrates Black Lives Matter

^a^ Twitter is now known as X.

^b^ Overlaps with vaccine safety theme.

#### 3.2.5 Philosophical/religious objections

The belief that vaccines contain substances that conflict with personal or religious beliefs, emerged from three sub-themes within the overarching theme of philosophical/religious objections. One sub-theme centred on the belief that vaccines contain foetal tissue, which raised concerns over the use of aborted babies in vaccine preparations. This sub-theme included conversations about refusing vaccination because of religious beliefs that are against abortion. The second sub-theme centred on the use of porcine products in vaccines, which conflicts with religious beliefs that are against the ingestion of pork. The third sub-theme centred on any type of animal products being used to manufacture vaccines, where vegans expressed concerns over the use of animal-based additives in vaccine formulations, while animal rights activists perceived the use of animal products as an act of cruelty against animals. A fourth sub-theme centred on mandatory vaccination being perceived as a violation of human rights, with examples from other countries being discussed. See Table 9 for examples of these posts.

**Table 9:** Examples of quotes expressing philosophical/religious objections.

Source	Verbatim quotes
Blog	.. were fired for refusing flu vaccines for religious reasons. They won an EEOC settlement of $300,000 which requires them to be reinstated and receive back pay and compensatory damages
Blog^a^	Is forcing a person to be vaccinated legal, ethical or rational?…how does stabbing someone with a syringe filled with a biological and inherently toxic substance—against his/her will—as a condition for keeping his/her job and livelihood in any way acceptable?
Twitter^b^	BOMBSHELL: Complete list of vaccine excipient ingredients approved by CDC (includes cells from aborted human foetus)
Twitter	Vaccine warning for VEGANS: Vaccines are made with a cocktail of animal parts, human foetal tissue cell lines and African monkey cells…
Twitter	NY court lets woman refuse vaccine made with aborted baby tissue…#orthodox #theology
Twitter	[Name] discusses why he believes HPV vaccines are a crime against children
Twitter	If you call yourself a Christian, or even a Muslim, then vaccines should not be an option for you
Twitter	Nurses Across the Country Standing Up For Their Right to Refuse Mandatory Vaccinations
Twitter	In Islam every part of the swine is haram. Vaccinations which contain porcine (pork) is also haram for Muslims. Do not vaccinate with haram
Twitter	Mandatory obedience: Hospitals are threatening the jobs of healthcare workers who refuse the flu vaccine

^a^ Overlaps with vaccine safety theme.

^b^ Twitter is now known as X.

## 4 Discussion

To the best of our knowledge, this study is the first to report on public sentiments about a broad range of vaccines expressed on South African social media platforms with unrestricted data access, providing evidence of relatively high levels of pre-existing negative sentiments toward vaccines in the pre-pandemic era. Our finding that more than 80% of vaccine-related posts were on Twitter is supported by a multi-national (15 countries, including South Africa) study on vaccination of pregnant women conducted from 1 November 2018–30 April 2019, where almost 90% of social media posts were on Twitter (34). However, it must be borne in mind that at the time of our study, Twitter was used by only 25% of South African social media users (35). Because our search was limited by social media platforms' privacy rules (a limitation that applies to all “social listening” projects, including the multi-national study on pregnant women) and the capabilities of Pulsar® software, Facebook, YouTube and Instagram (used by 49%, 47% and 25% of South African social media users, respectively) were excluded from our study. Our finding that 9.7% of posts mentioning specific vaccines expressed negative sentiments is in line with the South African results of this multinational study, which reported that 8.1% of South African posts about maternal vaccination were negative (34). However, our finding that 16.2% of posts overall expressed negative sentiments indicates that hesitancy toward vaccines is often more general and not confined to any specific vaccine, because of a lack of trust in health authorities, which has been identified as a major driver of vaccine hesitancy (3). This may help explain why, in the post-pandemic era, COVID-19 vaccine hesitancy may also negatively impact the uptake of infant and other vaccines that were accepted without questioning in the pre-pandemic era, as previously suggested (27, 28, 31).

The multinational study of maternal vaccination reported South Africa as one of three countries (South Korea and Germany being the other two) most affected by “discouraging” tweets originating in the USA (34), which supports our finding of the predominance of links to anti-vaccination articles emanating from the USA. This observation also echoes findings of an earlier study on anti-vaccination lobbying on the South African internet, which reported that from 2011–2013, 71.6% of anti-vaccination content originated from the USA alone, while a further 6.0% originated from both the USA and the United Kingdom (UK) (32).

The social media vaccine discourse during our study period was dominated by four vaccines. Unfortunately, sub-optimal vaccination coverage had resulted in a major measles outbreak in South Africa throughout 2017, at the same time that our study was conducted (36). This may explain why 19.4% of posts were about the measles vaccine, while the finding that almost 95% of measles vaccine-related posts were positive may be explained by the social media campaigns promoting measles vaccination conducted by the South African National Department of Health (NDoH) and Provincial Departments of Health (DoH) and their partners throughout the outbreak. Following close behind, the HIV vaccine was the topic of 19.1% of posts, with almost 95% being positive. This was most likely because a highly publicised clinical trial to test the safety and efficacy of an experimental HIV vaccine was launched just before we started our study, with participants being recruited throughout the duration of our study (37). Only two other vaccines were discussed in more than 10% of posts in our study—those against influenza (13.9% of posts) and HPV (12.4% of posts). Their relative prominence during the period when our study was conducted is most likely because every year (a) the annual influenza vaccination campaigns start in March (38), and (b) annual school-based HPV vaccination campaigns are held in February and March. Thus, social mobilisation campaigns conducted by the NDoH, Provincial DoH and their partners using all types of media, including social media, promote these vaccines during February and March every year, which also explains why the majority of comments about these vaccines were positive, despite both vaccines attracting the largest numbers of negative comments. This latter finding was unsurprising, given that those expressing negative sentiments in our study relied heavily on articles predominantly emanating from the USA, where vaccine hesitancy toward these two vaccines has been found to be higher than toward routine childhood vaccines (39–41).

Except for the measles vaccine, all other vaccines given routinely during early childhood were discussed in less than 10% of posts. However, negative sentiments were expressed in more than 10% of posts discussing most of these vaccines, namely the combined measles mumps rubella vaccine (MMR) (39.6% negative); and those against chickenpox (38.9% negative), polio (17.4% negative), tetanus (15.4% negative), mumps (12.7% negative), hepatitis (11.4% negative), tuberculosis (11.4% negative) and pertussis (11.1% negative). Of interest is that MMR and varicella vaccines against chickenpox are only available through the South African private sector. Furthermore, caregivers utilising private sector healthcare services represent only 16% of the South African population, and are much more affluent than the 84% utilising the public sector (7). Thus, our finding that almost 40% of posts about these two private sector vaccines were negative supports the suggestion made in the introduction, that online and social media research in South Africa is more likely to report higher levels of vaccine hesitancy than face-to-face household surveys, since relatively wealthy gated communities are seldom accessed during these surveys. In addition, the extent of negative sentiment expressed towards MMR was expected, because the discredited claim that MMR causes autism continues to circulate globally (42) and has been evident on South African webpages since at least 2011 (32). This claim originated from unethical, fraudulent, discredited research published in 1998 and subsequently retracted in 2010 (43), authored by a doctor from the UK who has since emerged as a leader of the modern anti-vaccination movement in the USA (44). Also, at the time our study was conducted, a tetravalent vaccine against MMR and varicella (MMRV) was available in the South African private sector. It is thus possible that the varicella vaccine may have become associated with MMR in public discourse, thus attracting a relatively large proportion of negative sentiment.

Our identification of vaccine safety concerns as a major theme was not unexpected. Since the first vaccine against smallpox was introduced in the early 19th century, vaccine safety has always been a major theme driving vaccine hesitancy globally (42, 45), with the Wellcome Global Monitor 2018 reporting that 7% of over 140,000 participants from 140 countries somewhat or strongly disagreed with the statement “vaccines are safe”, while 9% of South Africans disagreed (46). Vaccine safety concerns have also featured in pre-pandemic South African studies reporting on online content or reasons for not vaccinating, with three of these focusing on HPV vaccination, which since 2014 have been available free of charge to girls aged ≥9 years attending South African public sector schools (9–11). First, an online survey of caregivers of girls attending private sector schools in 2018 reported that only 19.4% of age-eligible girls had received ≥1 HPV vaccine dose, with 28.8% of caregivers of unvaccinated girls being concerned about vaccine safety (11). Second, a hard-copy self-administered questionnaire-based survey of caregivers of girls attending public sector schools in a district of South Africa in 2019 where ≥1dose HPV vaccination coverage was found to be 67.1%, reported that 31.9% of caregivers of unvaccinated girls were concerned about vaccine safety, while 15.1% had heard rumours of serious adverse events following immunisation [AEFI] with the HPV vaccine (10). Third, an analysis of comments in response to a February 2019 announcement on the Western Cape DoH's Facebook page about the commencement of the annual HPV vaccination campaign, identified 33% as negative, with vaccine safety concerns being a major driver (9). Only one study was based on infant vaccination, which reported that 95% of websites publishing anti-vaccination content from 2011–2013 claimed that vaccines are not safe, while 73.1% claimed that the risk of AEFI was higher than the risk of the disease itself (32). The multi-national study of 15 countries mentioned previously, was a discourse analysis based on social media posts regarding maternal vaccination (34). This study reported that South Africa was one of three countries (USA and Italy being the other two) expressing safety concerns about maternal vaccination, with posts reflecting fear and anger related to the perception that maternal vaccination results in foetal death and various diseases (34).

While autism concerns related to vaccination are a type of vaccine safety concern, autism concerns emerged as a major theme in our study, with some posts also expressing conspiracy theories. For the same reason we expected a high proportion of negative sentiment towards MMR, we were unsurprised that autism concerns emerged as a major theme in our study. However, the MMR did not emerge within this theme in our study, where vaccines in general (particularly “excessive” quantities of vaccines, combined vaccines and mercury in vaccines) were blamed for causing autism. This finding is supported by the multinational study on maternal vaccination, where autism was linked to all maternal vaccinations, with South Africa being one of three countries (USA and Italy being the other two) expressing these concerns (34).

Similar to vaccine safety concerns, concerns about vaccine effectiveness also have their roots in objections to the introduction of smallpox vaccination in the 19th century. At that time, causal analysis using inferential statistical methods had not yet been invented, and Alfred Russel Wallace, a scientist who, along with Charles Darwin, had discovered the theory of evolution, based his stance against mandatory smallpox vaccination on the lack of scientific evidence of its effectiveness (45). While actuarial statistical analysis of life tables and smallpox mortality rates was used by both pro- and anti-vaccination scientists at that time, his interpretation of the data led him to conclude that smallpox mortality rates increased as smallpox vaccination coverage increased, while pro-vaccination scientists concluded the exact opposite (45). Since that time, so-called science-based claims of lack of vaccine effectiveness are often based on inappropriate simplistic statistical analysis, despite overwhelming evidence of the effectiveness of vaccines from studies using appropriate inferential statistical analysis (32). The Wellcome Global Monitor 2018 reported that 5% of participants globally somewhat or strongly disagreed with the statement “vaccines are effective”, while 11% of South Africans disagreed (46), perhaps because South Africans are more affected by vaccine misinformation originating in other countries, as shown elsewhere (34). The emergence of this claim as a major theme in our study is also in agreement with previous South African internet-based and social media research, with 65.7% of South African websites publishing anti-vaccination content from 2011–2013 making this claim (32), while the perception that there is no evidence of HPV vaccination preventing cervical cancer was expressed by Facebook users in response to the announcement of the 2019 HPV vaccination campaign (9). Similarly, 4.4% and 10.5% of caregivers of unvaccinated age-eligible girls attending private and public sector schools respectively in South Africa, perceived that the HPV vaccine was ineffective (10, 11).

The spread of conspiracy theories fuelled vaccine hesitancy globally long before the COVID-19 pandemic (4, 42, 47) and featured on South African webpages since at least 2011 (32). While conspiracy theories on South African webpages discrediting vaccines from 2011–2013 focused on the profit motive of pharmaceutical companies (32), a much broader range of conspiracy theories were mentioned within the theme that emerged from our study. Many of these posts overlapped with the vaccine safety concern theme and reflected distrust in government, the pharmaceutical industry, and local and global health authorities. Furthermore, posts centering on racial motives against Africans reflected distrust emanating from past and present social injustices inflicted on Black South Africans. These findings provide further evidence that social, cultural, historical, institutional and political factors unrelated to vaccination are driving vaccine hesitancy globally, as previously posited by others (4, 47).

Philosophical and religious objections to vaccination are also rooted in the introduction of smallpox vaccination in the 19th century, in response to laws enforcing mandatory vaccination. Widespread anti-vaccination lobbying resulted in a law being passed in the UK in 1898, allowing for exemptions based on conscientious objection to vaccination (45). In the USA, even in states where smallpox vaccination was not mandatory, conscientious objections to vaccination centred on the violation of civil rights and personal freedoms, including religious freedom (48). It has previously been shown that vaccination also poses ethical and religious concerns for some sectors of South African society (32), which supports our findings of negative sentiments toward vaccination being expressed by vegans, animal rights activists and those with religious beliefs that conflict with vaccination. Also, despite vaccination not being mandatory in South Africa, mandatory vaccination as a violation of human rights emerged as a sub-theme in our study, which again supports previous findings of the influence on South African social media users exerted by negative posts emanating from the USA (34).

## 5 Limitations

Like most online “social listening” platforms, Pulsar® uses IP addresses to geolocate the origin of content. However, the global use of virtual private networks (VPNs), including in South Africa and the rest of Africa (https://www.cmcnetworks.com/africa-vpn-services.html), limits the validity of these data. This is because VPN users from outside South Africa who used South African VPNs, would have been included in the dataset. Also, South Africans using VPNs from other countries would have been excluded from the dataset. This is an unavoidable limitation which to the best of our knowledge is experienced by all researchers engaged in online “social listening” projects.

Six months may not have been long enough to fully establish pre-pandemic public sentiment, but given the time-consuming and labour-intensive methods used for analysing the data where more than 85% of software-assigned sentiments had to be changed, it was necessary to confine our study to a six-month period. During this time, social media was used by the NDoH to promote measles, HPV and influenza vaccines, thus it is possible that there may have been an over-representation of positive sentiments being expressed toward these vaccines in our study.

The finding that most posts were predominantly written in English was expected, since it has been shown that most South African indigenous language speaking social media users prefer to post in English, interspersed with words in indigenous languages (33). While we did not record which languages were mixed with English in each post, switching between languages when speaking is common in South Africa, which is a multilingual country where most people are able to speak more than one language (33). However, it is possible that we may have missed some posts where indigenous language words instead of vaccine were used, thus we acknowledge this as a possible limitation, although these words were not found in previous South African online research (32).

Since our study was limited by social media platforms' privacy rules and the capabilities of Pulsar® software, our results are biased towards the views of South African social media users who were active on Twitter at the time of our study. Since there is evidence of distinct differences in the socio-demographic profiles of users of different social media platforms in the USA (49), it is likely that the same is true for South African social media users. While data from South Africa are lacking, it can be assumed that this is the most important limitation of our study.

## 6 Conclusions

This study provides pre-pandemic evidence of a relatively high proportion of vaccine hesitancy being expressed on South African social media, with 16.2% of posts during the study period expressing negative sentiments toward vaccination, including vaccines used for routine childhood immunisation, with negative sentiment proportions reaching almost 40% for some infant vaccines, while HPV vaccines attracted the most negative sentiments overall. The Vaccine Confidence Project™ previously identified social media monitoring as a method for early detection of waning levels of public confidence in vaccines, allowing health authorities to respond with timely, targeted and appropriate interventions (5). While the methods we used in our study were time-consuming, a scoping review of vaccine-related social media monitoring methods conducted in 2018, identified 20 studies using automated monitoring tools (50). While further research is needed to identify the most accurate and affordable automated tool to use in our setting, the results of this study can be used as a baseline against which future studies can be compared.

## Data Availability

The original contributions presented in the study are included in the article/Supplementary Material, further inquiries can be directed to the corresponding author.
